# Hrk1 Plays Both Hog1-Dependent and -Independent Roles in Controlling Stress Response and Antifungal Drug Resistance in *Cryptococcus neoformans*

**DOI:** 10.1371/journal.pone.0018769

**Published:** 2011-04-13

**Authors:** Seo-Young Kim, Young-Joon Ko, Kwang-Woo Jung, Anna Strain, Kirsten Nielsen, Yong-Sun Bahn

**Affiliations:** 1 Department of Biotechnology, College of Life Science and Biotechnology, Center for Fungal Pathogenesis, Yonsei University, Seoul, Korea; 2 Department of Microbiology, Medical School, University of Minnesota, Minneapolis, Minnesota, United States of America; Research Institute for Children and the Louisiana State University Health Sciences Center, United States of America

## Abstract

The HOG (High Osmolarity Glycerol response) pathway plays a central role in controlling stress response, ergosterol biosynthesis, virulence factor production, and differentiation of *Cryptococcus neoformans*, which causes fatal fungal meningoencephalitis. Recent transcriptome analysis of the HOG pathway discovered a Hog1-regulated gene (CNAG_00130.2), encoding a putative protein kinase orthologous to Rck1/2 in *Saccharomyces cerevisiae* and Srk1 in *Schizosaccharomyces pombe*. Its function is not known in *C. neoformans*. The present study functionally characterized the role of Hrk1 in *C. neoformans*. Northern blot analysis confirmed that *HRK1* expression depends on the Hog1 MAPK. Similar to the *hog1Δ* mutant, the *hrk1Δ* mutant exhibited almost complete resistance to fludioxonil, which triggers glycerol biosynthesis via the HOG pathway. Supporting this, the *hrk1Δ* mutant showed reduced intracellular glycerol accumulation and swollen cell morphology in response to fludioxonil, further suggesting that Hrk1 works downstream of the HOG pathway. However, Hrk1 also appeared to have Hog1-independent functions. Mutation of *HRK1* not only further increased osmosensitivity of the *hog1Δ mutant*, but also suppressed increased azole-resistance of the *hog1Δ* mutant in an Erg11-independent manner. Furthermore, unlike the *hog1Δ* mutant, Hrk1 was not involved in capsule biosynthesis. Hrk1 was slightly involved in melanin production but dispensable for virulence of *C. neoformans*. These findings suggest that Hrk1 plays both Hog1-dependent and –independent roles in stress and antifungal drug susceptibility and virulence factor production in *C. neoformans*. Particularly, the finding that inhibition of Hrk1 substantially increases azole drug susceptibility provides a novel strategy for combination antifungal therapy.

## Introduction

The HOG (High Osmolarity Glycerol response) pathway plays central roles in several cellular functions in filamentous and non-filamentous fungi, encompassing both pathogens and non-pathogens. This pathway is not only important in maintaining normal cellular homeostasis under a plethora of environmental stresses, but is also required for development and differentiation of fungi (see reviews, [1], [2]). Particularly for pathogenic fungi, the HOG pathway is one of the major signaling cascades for controlling production and regulation of virulence factors [2]. The mammalian counterpart for the fungal HOG pathway is the p38 MAPK pathway, which also plays a pivotal role in stress response, immunity modulation, and cell death [3], [4].

The basidiomycetous fungus, *Cryptococcus neoformans*, causes fatal lung and brain infections if left untreated. The HOG pathway in *C. neoformans* is one of the major signaling pathways to control stress response, differentiation, and virulence factor production and is structurally similar to those found in other fungi. External signals are sensed and transferred by the two-component-like phosphorelay system, which consists of hybrid sensor histidine kinases, named Tco (Two-component-like) proteins, the histidine-containing phosphotransfer (HPt) protein, Ypd1, and two response regulators, Ssk1 and Skn7. Downstream of the phosphorelay system, Ssk1 further activates a mitogen-activated protein kinase (MAPK) module, which comprises a Hog1 MAPK, Pbs2 MAPK kinase (MAPKK), and Ssk2 MAPKK kinase (MAPKKK). This signaling cascade controls cellular responses against a variety of environmental cues, including osmotic shock, high temperature, oxidative stress, UV irradiation, heavy metal, antifungal drugs, and toxic metabolites [2], [5], [6], [7], [8], [9]. The Skn7 response regulator is also involved in Na^+^ stress response, oxidative stress response, and antifungal drug resistance, but mostly in a Hog1-independent manner [6].

Regardless of the common features of the *Cryptococcus* HOG pathway, the pathway has undergone unique specialization in terms of regulation and cellular functions. Most notably, the Hog1 MAPK is constitutively phosphorylated under unstressed conditions in a number of clinical and environmental *C. neoformans* strains, including strain H99 (serotype A genome sequencing platform strain) and B-3501A (serotype D genome sequencing platform strain), which is in stark contrast to most fungi, where Hog1 is unphosphorylated under normal conditions but phosphorylated in response to environmental stresses [5], [8]. The constitutive phosphorylation of Hog1 appears to provide unique phenotypes to the HOG mutants in *C. neoformans*, including increased production of two important virulence factors of *C. neoformans*, antiphagocytic capsule and antioxidant melanin, and enhanced sexual differentiation [5], [6], [8]. The Ssk2 MAPKKK has been identified as a major upstream signaling component that is responsible for constitutive phosphorylation of the Hog1 MAPK through comparative analysis of meiotic maps between the serotype D f1 sibling strains B-3501 and B-3502, which show differential Hog1 phosphorylation patterns [8]. Two nonsynonymous coding sequence changes (L240F and M738V) in the N-terminal region of Ssk2 between strains B-3501 and B-3502 appear to define the different phosphorylation patterns of Hog1 [8].

To identify downstream target genes and to clarify the complex signaling network of the *Cryptococcus* HOG pathway, comparative transcriptome analysis has recently been performed, revealing a number of novel characteristics of the pathway [9]. First, the HOG pathway negatively controls expression levels of ergosterol biosynthesis genes, including *ERG11*, resulting in increased cellular ergosterol content when the *HOG1*, *PBS2*, *SSK2*, or *SSK1* genes are mutated. Therefore, mutation of the HOG pathway components confers dramatic synergistic antifungal effects with amphotericin B, which binds to the ergosterol of fungal cell membranes and forms a transmembrane channel, causing a leakage of essential ions and eventually cell death. Increased cellular ergosterol content by inhibition of the HOG pathway is likely to provide more binding targets for the polyene drug and thereby enhance its antifungal efficacy. Furthermore, a number of cadmium stress-responsive genes are differentially regulated and indeed the HOG mutants show increased resistance to cadmium treatment. These features of the *Cryptococcus* HOG pathway have not been described previously in other fungi.

This prior transcriptome analysis discovered several hitherto uncharacterized protein kinases, whose expression appears to be differentially regulated by the *Cryptococcus* HOG pathway [9]. Among these we have identified a gene, (CNAG_00130.2), which has greatly decreased basal expression in both *ssk1Δ* and *hog1Δ* mutants. CNAG_00130.2 shows significant homology to Hog1-dependent MAPK-activated protein kinases (MAPKAPKs) in other fungi. In *S. pombe*, two MAPKAPKs, Srk1 (also known as Mkp1) and Cmk2 (also known as Mkp2), have been reported to physically interact with and be activated via phosphorylation by the Sty1 MAPK (Hog1 homologue) [10], [11]. Expression of *SRK1* is induced by a range of environmental stimuli, including oxidative or osmotic stress, and heat shock, in a Sty1-dependent manner [11]. Srk1 is involved in conjugation, meiosis, and sporulation [11] whereas Cmk2 modulates the oxidative stress response [10]. Srk1 is also required for regulation of *S. pombe* cell cycle progression [12]. In *S. cerevisiae*, two MAPKAPKs, Rck1 and Rck2, have been discovered and between these Rck2 is activated by phosphorylation via the Hog1 MAPK [13], [14], [15]. Although the *rck1Δ*, *rck2Δ*, and *rck1Δ rck2Δ* mutants do not show any distinctive phenotypes, both Rck1 and Rck2 are involved in oxidative stress response and meiosis [16], [17].

In this study we confirmed that expression of CNAG_00130.2 is controlled by the Hog1 MAPK in *C. neoformans* and thereby named it Hrk1 (Hog1-regulated kinase 1). To functionally characterize Hrk1, we constructed targeted deletion mutants for *HRK1* in both wild-type and *hog1Δ* mutant strain backgrounds and performed an array of phenotypic analyses in comparison with the *hog1Δ* mutant. Our data suggest Hrk1 plays both Hog1-dependent and –independent roles in controlling stress and antifungal drug response and production of virulence factors, such as melanin, in *C. neoformans*. However, Hrk1 is dispensable for virulence of *C. neoformans*. Notably, mutation of *HRK1* not only suppressed azole-resistance of the *hog1Δ* mutant, but also further increased azole-sensitivity compared to the wild-type, strongly suggesting a novel drug target for treatment of cryptococcosis.

## Results

### Identification of Hog1-regulated kinase 1 (Hrk1) in *C. neoformans*

**Figure 1:**
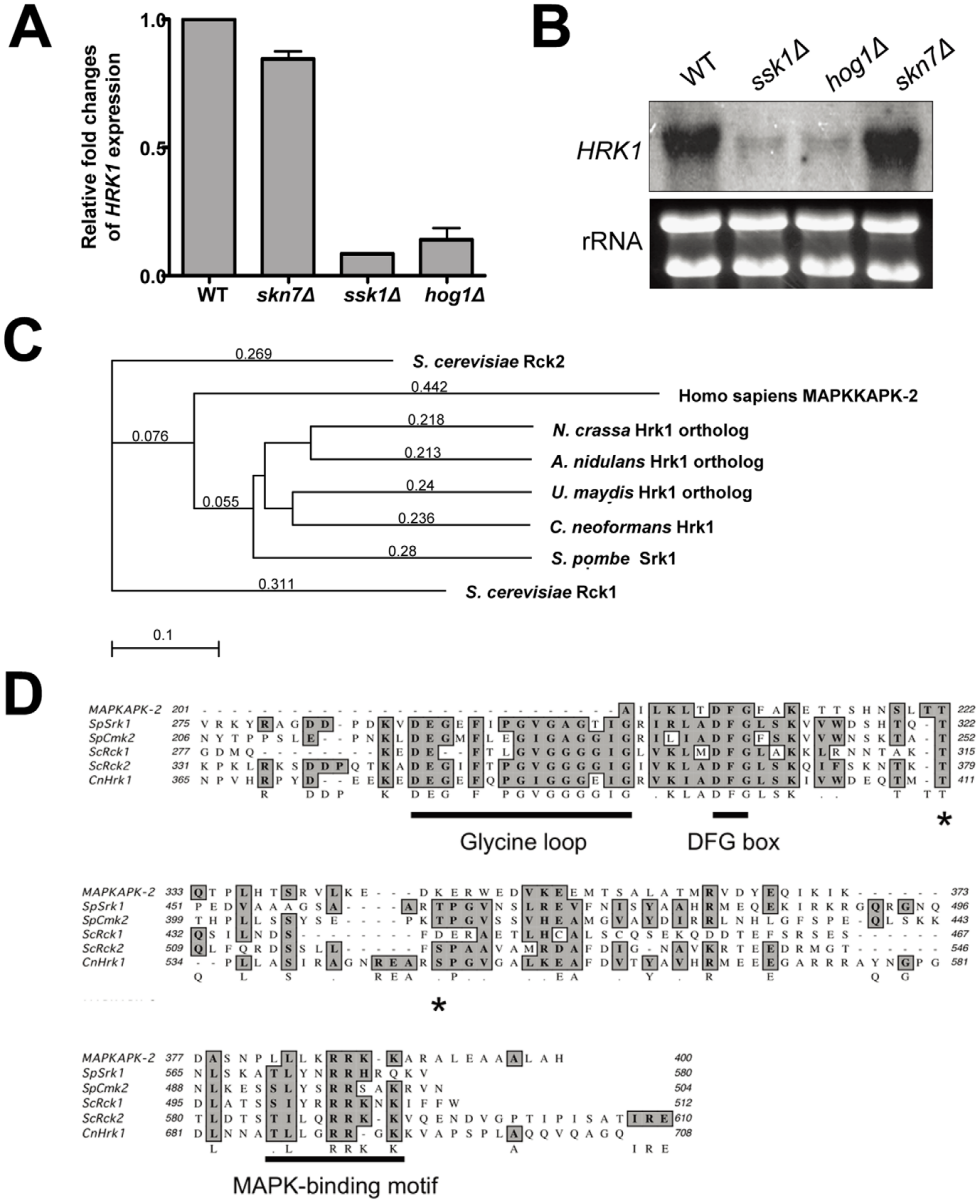
Identification of Hog1-regulated kinase, Hrk1, in *C. neoformans*. (A) The relative expression levels of the *HRK1* gene (CNAG_00130.2) from microarray data using total RNA isolated from the WT (H99) strain and *ssk1Δ*, *skn7Δ*, and *hog1Δ* mutants grown to the middle logarithmic phase at 30°C in YPD medium [9]. (B) Northern blot analysis for measuring basal expression levels of *HRK1* using total RNA of (A). (C) The phylogenetic analysis of Hrk1 orthologs between *C. neoformans* and other eukaryotes. The following proteins were compared for the analysis: *S. cerevisiae* Rck1 (YGL158W, SGDID: S000003126), *S. cerevisiae* Rck2 (YLR248W, SGDID: S000004238), Homo sapiens MAPKAPK-2, *N. crassa* Hrk1 (NCU09212.4), *A. nidulans* Hrk1 ortholog (AN4483, AspGDID: ASPL0000072431), *U. maydis* Hrk1 ortholog (UM02121), *C. neoformans* Hrk1 (CNAG_00130.2) and *S. pombe* Srk1 (SPCC1322.08). The phylogenetic tree was generated by CLUSTAL W (tree-building method, neighbor joining) with MacVector software (version 7.2.3; Accelrys). (D) Multiple sequence alignment of Hrk1 orthologs is depicted by Clustal W alignment from MacVector software.

The prior transcriptome analysis for elucidation of the downstream signaling network governed by the HOG pathway revealed that a *C. neoformans* gene (CNAG_00130.2), which encodes a putative member of the Ca^2+^/calmodulin-dependent protein kinase superfamily, is significantly downregulated even under unstressed condition in both *ssk1Δ* and *hog1Δ* mutants, but not in the *skn7Δ* mutant [9] (Fig. 1A). To further confirm whether this gene is truly regulated by the HOG pathway, we performed Northern blot analysis. In agreement with the microarray data, basal expression levels of CNAG_00130.2 were significantly lower in both *hog1Δ* and *ssk1Δ* mutants than the wild-type and *skn7Δ* mutant strains (Fig. 1B), strongly indicating that it is indeed differentially regulated by the HOG pathway in *C. neoformans*. Therefore the CNAG_00130.2 was named Hrk1 (Hog1-regulated kinase 1).

*C. neoformans* Hrk1 is highly homologous to *S. pombe* Srk1/Mkp1, *S. cerevisiae* Rck2, and mammalian MAPKAPK-2, all of which are known to be p38/Hog1 MAPK-dependent protein kinases [11], [15], [16], [18](Fig. 1C). Hrk1 is more homologous to Srk1 (52% identity in amino acid sequence) than Rck2 (45% identity) and MAPKAPK-2. Hrk1 shares several structural features with the yeast Hog1/Sty1-dependent MAPKAPKs. First, the glycine-rich domain (also known as the glycine-loop) and the highly conserved DFG box in the kinase catalytic domain were also observed in Hrk1 (Fig. 1D). Second, a putative MAPK-binding motif with a consensus RR sequence observed in other MAPKAPKs was also found in Hrk1 (Fig. 1D). The serine residue (Ser519) of Rck2, which is known to be activated by phosphorylation, was also conserved in Hrk1, although the Ser residue appears not to be conserved in other MAPKAPKs. Indeed the Thr411 in Cmk2 was found to be in the position equivalent to Ser519 residue in Rck2 and to be essential for phosphorylation by Sty1 for its activation [10].

### *HRK1* expression is differentially regulated in response to environmental cues via the Hog1 MAPK

**Figure 2:**
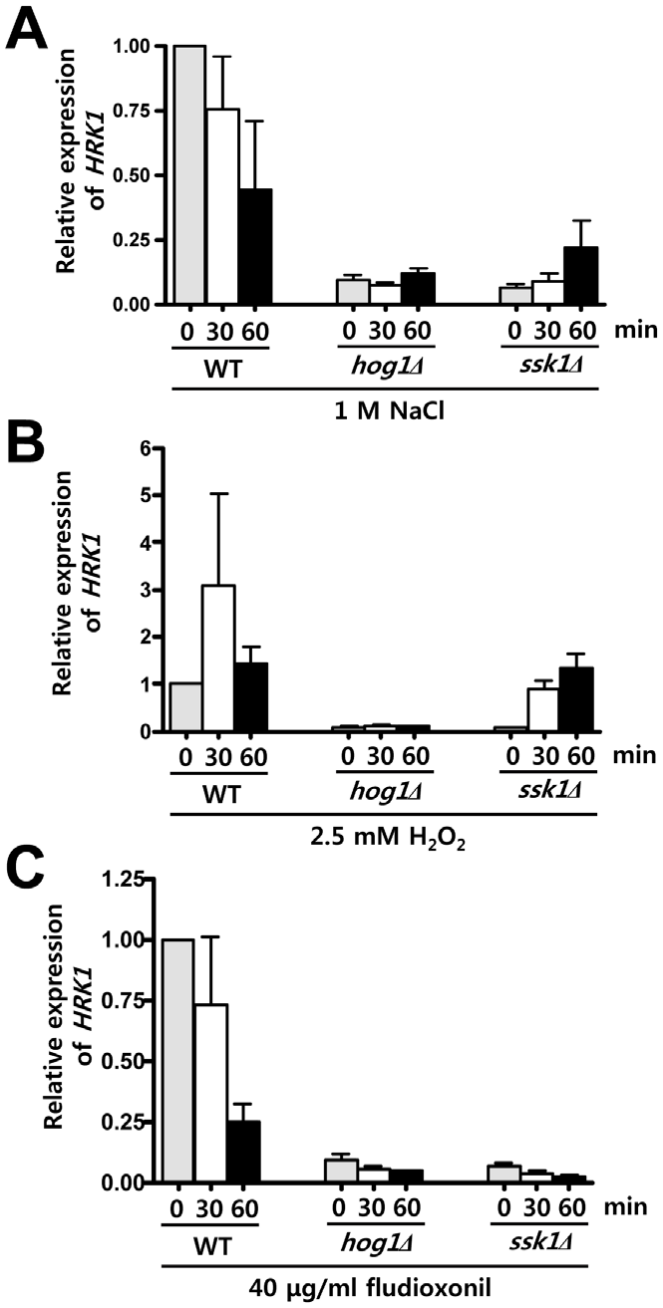
Analysis of stress-dependent expression patterns of *HRK1* in *C. neoformans*. Total RNA was isolated from WT and the *ssk1Δ* and *hog1Δ* mutants grown in YPD medium containing 1 M NaCl for osmotic stress (A), 2.5 mM H_2_O_2_ for oxidative stress (B), or 40 µg/ml fludioxonil for antifungal drug treatment (C) at different time points (0, 30 and 60 min). For quantitative RT-PCR (qRT-PCR), data collected from three independent biological replicates with three technical replicates were normalized by using *ACT1* as a control. Relative gene expression indicates *HRK1* expression levels of each strain and time point compared to those of the wild-type strain at zero time point (unstressed condition).

We examined whether *HRK1* expression is modulated in response to environmental stress, such as osmotic/salt shock, oxidative stress, and fludioxonil treatment since the HOG pathway is one of major stress-activated signaling pathways in *C. neoformans*. The quantitative RT-PCR revealed that *HRK1* expression was downregulated in the wild-type strain in response to 1 M NaCl (Fig. 2A). In the *hog1Δ* mutant, expression levels of *HRK1* were generally low under both unstressed and osmotically stressed conditions (Fig. 2A). Interestingly, basal expression levels of *HRK1* were also very low in the *ssk1Δ* mutant, but its expression increased at the later time point (60 min) during NaCl treatment (Fig. 2A), indicating that *HRK1* expression needs to be induced in response to osmotic shock in the absence of the Ssk1 response regulator, but requires the Hog1 MAPK.

In response to hydrogen peroxide (2.5 mM H_2_O_2_), *HRK1* expression was upregulated in the wild-type strain. Both basal and induced levels of *HRK1* were also generally low in the *hog1Δ* mutant (Fig. 2B). Similar to the results seen during the osmotic stress response, however, *HRK1* expression levels were strongly induced after 30 min of exposure to H_2_O_2_ in the *ssk1Δ* mutant and became equivalent to those of the wild-type strain (Fig. 2B). These data indicate that Hrk1 expression can be induced by H_2_O_2_ in an Ssk1-independent but Hog1-dependent manner. In response to fludioxonil, however, both basal and induced levels of *HRK1* were significantly lower in both the *hog1Δ* and *ssk1Δ* mutants than in the wild-type strain (Fig. 2C). Northern blot analysis also displayed similar results (Fig. S1). Interestingly, *HRK1* expression levels appear to be roughly proportional to the level of phosphorylated Hog1. In the H99 strain background, Hog1 is highly, constitutively phosphorylated under unstressed conditions [5], which may reflect high *HRK1* basal expression levels. In response to NaCl or fludioxonil, Hog1 phosphorylation levels decrease [5], [6], which is similar to NaCl or fludioxonil-induced reduction of *HRK1* expression. Also the Ssk1-independent induction of *HRK1* presumably reflects the Ssk1-independent phosphorylation of Hog1 in response to NaCl [6]. In conclusion, basal *HRK1* expression appears to be mainly controlled by the Ssk1 response regulator and the Hog1 MAPK. In response to osmotic and oxidative stresses, however, *HRK1* can be induced via Hog1 by an unknown signaling component, other than Ssk1.

### Hrk1 regulates osmotic stress response and fludioxonil susceptibility by controlling intracellular glycerol accumulation via the Hog1 MAPK

To explore the function of Hrk1, the *HRK1* gene was disrupted by targeted deletion and comparative mutant analysis with other HOG mutants was performed. The *hrk1Δ* mutant allele was generated by overlap PCR and biolistically transformed into the wild-type *C. neoformans* serotype A H99 (*MAT*α) and KN99**a** (*MAT* **a**) strains as described in Materials and Methods. Three independent *hrk1Δ* mutants (YSB270, YSB271, and YSB272) were generated in the *MAT*α background and confirmed by Southern blot analysis (Fig. S2). For verification of any phenotypes of the *hrk1Δ* mutant, a complemented strain (*hrk1Δ*+*HRK1*) was constructed by re-integrating the wild-type *HRK1* into its native locus. Furthermore, to address whether Hog1 is the only upstream MAPK for regulating expression and activity of Hrk1 and which Hog1 processes are likely mediated through Hrk1, we also constructed the *hog1Δ hrk1Δ* double mutant (Fig. S3). If Hog1 is the only upstream regulator of Hrk1, the *hrk1Δ hog1Δ* mutant should exhibit phenotypes similar to the *hog1Δ* mutant.

**Figure 3:**
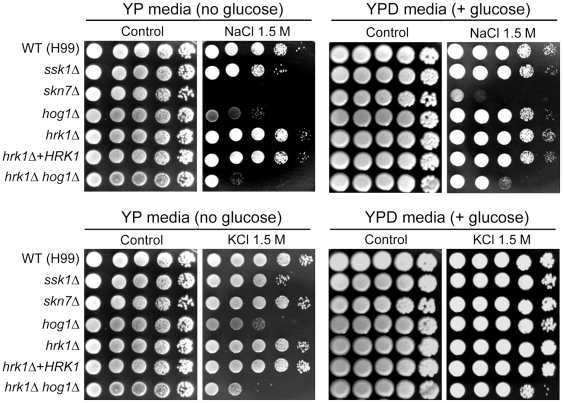
The role of Hrk1 in osmotic stress response of *C. neoformans*. Each *C. neoformans* strain was grown overnight at 30°C in liquid YPD medium, 10-fold serially diluted (1–10^4^ dilutions), and spotted (4 µl of dilution) on YPD (+glucose) or YP (−glucose) agar containing 1.5 M NaCl and 1.5 M KCl. Cells were incubated at 30°C for 72 h and photographed.

Supporting that Hrk1 is positioned downstream of Hog1, disruption of *HRK1* did not affect Hog1 phosphorylation patterns observed in the wild-type strain (Fig. S4). Next, we compared a variety of stress response phenotypes of the *hrk1Δ* mutant with those of *hog1Δ* and *hrk1Δ hog1Δ* mutants. First, we examined osmotic sensitivity of the *hrk1Δ* mutant, since Hog1 plays a role in maintaining osmotic balance, particularly under carbon starved conditions [5], [6]. The *hrk1Δ* mutant displayed wild-type levels of osmosensitivity under both glucose-rich and –starved conditions (Fig. 3). Interestingly, however, the *hrk1Δ hog1Δ* double mutant exhibited greater osmosensitivity to 1.5 M NaCl or KCl than the *hog1Δ* mutant (Fig. 3), suggesting that Hrk1 plays a minor role in maintaining osmotic balance in a Hog1-independent manner.

**Figure 4:**
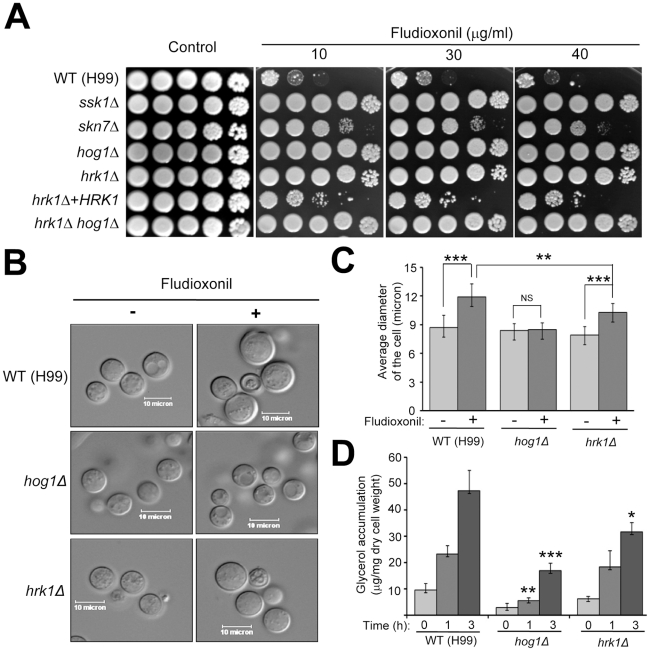
The role of Hrk1 in fludioxonil susceptibility and intracellular glycerol synthesis of *C. neoformans*. (A) Each strain grown was 10-fold serially diluted (1–10^4^ dilutions), and spotted (4 µl of dilution) on YPD agar containing indicated concentrations of fludioxonil. Cells were incubated at 30°C for 72 h and photographed. (B–C) Each strain was grown to the middle logarithmic phase in liquid YPD medium, reincubated in YPD or YPD medium containing fludioxonil (10 µg/ml) at 30°C for 48 hrs with shaking, and then photographed (B). Diameter of each strain treated with or without fludioxonil was quantitatively measured by using SPOT image analysis software (Diagnostic Instrument Inc.). The Y-axis indicates average diameter of the cell (µm, micron). (D) To quantitatively measure accumulation of intracellular glycerol content, each strain grown as described in Fig. 4B was reincubated in YPD containing fludioxonil (10 µg/ml) for the indicated incubation time. Glycerol content in cell extracts was measured by a UV-glycerol assay kit as described in Materials and Methods. Three independent experiments were performed. Standard deviations are presented as error bars. Statistical differences in relative cell diameter (C) or intracellular glycerol content (D) between strains were determined by Bonferroni's multiple comparison test. Each symbol indicates the following: *, *P*<0.05; **, *P*<0.01; ***, *P*<0.0001; NS, not significant (*P*>0.05).

Next, we monitored fludioxonil sensitivity of the *hrk1Δ* mutant. Fludioxonil hyperactivates the HOG pathway, triggering over-accumulation of intracellular glycerol content, and causes defective cytokinesis and cell swelling, which eventually results in cell cycle arrest [7]. Therefore, deletion of *HOG1* confers complete resistance to fludioxonil. Similar to the *hog1Δ* mutant, the *hrk1Δ* mutant was highly resistant to fludioxonil, although the *hog1Δ* mutant was more resistant than the *hrk1Δ* mutant (Fig. 4A). Re-integration of the *HRK1* gene restored fludioxonil sensitivity of the *hrk1Δ* mutant. Under exposure to fludioxonil the wild-type strain showed marked cell swelling, indicative of intracellular glycerol over-accumulation (Fig. 4B and 4C). The *hrk1Δ* mutant showed less cell swelling in response to fludioxonil than the wild-type strain, although more cell swelling than the *hog1Δ* mutant. To further support these findings, measurement of intracellular glycerol content upon fludioxonil treatment revealed that the *hrk1Δ* mutant had much lower levels of intracellular glycerol content than the wild-type strain, albeit to a greater extent than the *hog1Δ* mutant. Collectively, these data indicate that Hrk1 is the major downstream component of the Hog1 MAPK pathway to relay fludioxonil-responsive signaling, but other minor signaling component(s) may exist downstream of the Hog1 MAPK to confer full fludioxonil sensitivity through control of glycerol synthesis.

Because mutants with defects in cell wall integrity, such as *cna1Δ*, *mpk1Δ*, and *ras1Δ*, are hypersensitive to fludioxonil [7], [19], we also examined the role of Hrk1 in maintaining cell wall integrity. In contrast to previously described mutants, the *hrk1Δ* mutant showed wild-type levels of sensitivity to SDS, Congo red, DTT, and high temperature (Fig. S5). Furthermore, *hrk1* mutation did not exacerbate the increased sensitivity of the *hog1Δ* mutant to high temperature and SDS (Fig. S5). Taken together, these data indicate that Hrk1 is not required for maintenance of cell wall integrity.

### Hrk1 is involved in oxidative stress response in a Hog1-independent manner

**Figure 5:**
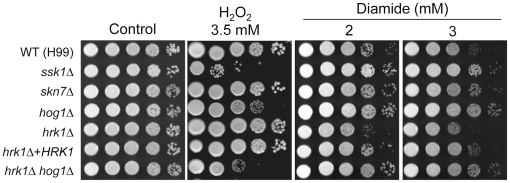
The role of Hrk1 in oxidative stress response of *C. neoformans*. Each *C. neoformans* strain was 10-fold serially diluted (1–10^4^ dilutions), and spotted (4 µl of dilution) on YPD agar containing the indicated concentration of diamide or hydrogen peroxide (H_2_O_2_). Cells were incubated at 30°C for 72 hrs and photographed.

The finding that *HRK1* expression is induced in response to H_2_O_2_ led us to examine the role of Hrk1 in the oxidative stress response of *C. neoformans*. *S. pombe* Srk1 and *S. cerevisiae* Rck2 are also known to be involved in oxidative stress response in a Sty1 or Hog1-dependent manner [16], [20]. In *C. neoformans*, the HOG pathway plays a critical role in defending against diverse oxidative damaging agents, such as H_2_O_2_, menadione, *tert*-butyl hydroperoxide (tBOOH), and diamide [5], [9]. The *hrk1Δ* mutant was clearly distinct from the *hog1Δ* mutant in oxidative stress response (Fig. 5). The *hrk1Δ* mutant showed wild-type levels of sensitivity to H_2_O_2_, menadione, and tBOOH, unlike the *hog1Δ* mutant that is hypersensitive to these agents (Fig. 5 and data not shown). However, the *hrk1Δ hog1Δ* mutant exhibited higher H_2_O_2_ sensitivity than the *hog1Δ* mutant, indicating that Hrk1 plays a minor role in oxidative stress response.

Diamide, which is not only an exogenous oxidant oxidizing thiol groups of proteins and glutathione but also a radiosensitizer perturbing DNA repair system, is known to confer oxidative stress response in a manner distinct from H_2_O_2_ [21]. As reported previously [19], the *hog1Δ* mutant showed increased resistance to diamide treatment (3 mM) compared to WT, which is in stark contrast to the results from H_2_O_2_ treatment (Fig. 5). Conversely, the *hrk1Δ* mutant was more sensitive to diamide than WT, which is in opposition to the pattern found in the HOG mutants (Fig. 5). Complementation with the WT *HRK1* gene restores normal diamide susceptibility to the *hrk1Δ* mutant, further confirming the involvement of Hrk1 in diamide resistance. Furthermore, mutation of *HRK1* decreased diamide-resistance of the *hog1Δ* mutant (Fig. 5). Taken together, these data suggest that *C. neoformans* differentially senses oxidative stress conferred by H_2_O_2_ and diamide, and that the HOG pathway and Hrk1 protein kinase appear to act on the oxidative stress response independently.

### Hrk1 controls azole resistance in a Hog1-independent manner

**Figure 6:**
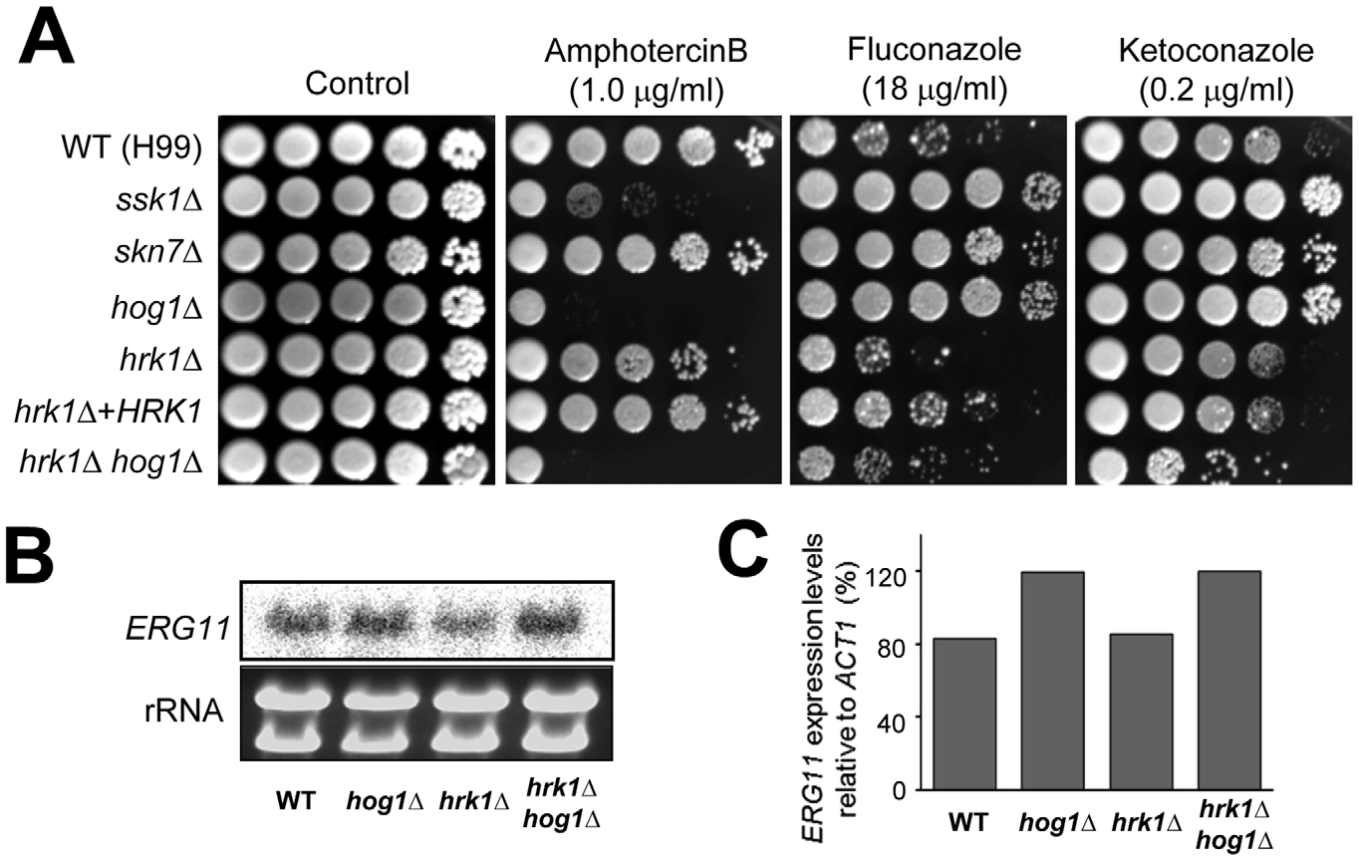
The role of Hrk1 in susceptibility to polyene and azole antifungal drugs of *C. neoformans*. (A) Each *C. neoformans* strain was 10-fold serially diluted (1–10^4^ dilutions), and spotted (4 µl of dilution) on YPD agar containing indicated concentrations of amphotericin B, fluconazole, or ketoconazole. Cells were incubated at 30°C for 72 hrs and photographed. (B–C) Northern blot analysis for examining expression profiles of *ERG11* in *hog1Δ*, *hrk1Δ*, and *hrk1Δ hog1Δ* mutants compared to the wild-type strain (H99) (B). The expression levels of *ERG11* relative to *ACT1* were measured via phosphorimager (Fuji BAS 2500) (C). The Y-axis indicates *ERG11* expression levels relative to *ACT1*, which is percent ratio of *ERG11* expression level vs. *ACT1* expression level. The *hog1Δ* and *hrk1Δ hog1Δ*, but not *hrk1Δ*, mutants showed higher *ERG11* expression levels compared to the wild-type strain.

The HOG pathway controls expression of ergosterol biosynthesis genes, including *ERG11*, and governs susceptibility to polyene and azole drugs. Accordingly, the HOG mutants exhibited increased sensitivity to the polyene class of antifungal agents, such as amphotericin B, but decreased sensitivity to the azole drugs, such as fluconazole and ketoconazole [9]. Increased ergosterol in the cell membrane by inhibition of the HOG pathway provides more binding sites for amphotericin B and enhances its antifungal activity whereas increased expression of *ERG11* requires higher concentration of azole drugs for its inhibition and thereby decreases their antifungal efficacy. To address whether Hrk1 controls sensitivity to polyene and azole drugs via the HOG pathway, we measured drug sensitivity of the *hrk1Δ* mutant. Compared to the *hog1Δ* mutant, the *hrk1Δ* mutant exhibited only a minor hypersensitivity to amphotericin B (Fig. 6A). However, the *hrk1Δ* mutant exhibited greater sensitivity to fluconazole than the wild-type strain whereas the *hog1Δ* mutant showed resistance (Fig. 6A). Notably, deletion of *HRK1* suppressed azole drug resistance of the *hog1Δ* mutant (Fig. 6A). The *hrk1Δ hog1Δ* mutant was even more sensitive to fluconazole and ketoconazole than the wild-type strain, implying that Hrk1 is involved in controlling azole sensitivity in a Hog1-independent manner.

To further verify these data, we examined expression levels of *ERG11*, the target gene of azole drugs, in the *hrk1Δ* mutant as *ERG11* expression is known to be induced in the *hog1Δ* mutant [9]. *ERG11* expression was indeed increased in the *hog1Δ* mutant compared to the wild-type strain as reported before, but not in the *hrk1Δ* mutant (Fig. 6B and 6C). Furthermore, *hrk1Δ* mutation did not further affect *ERG11* expression in the *hog1Δ* mutant. These data suggest that Hrk1 controls polyene and azole drug sensitivity in a Hog1-independent manner.

### The role of Hrk1 in virulence factor production and differentiation of *C. neoformans*

**Figure 7:**
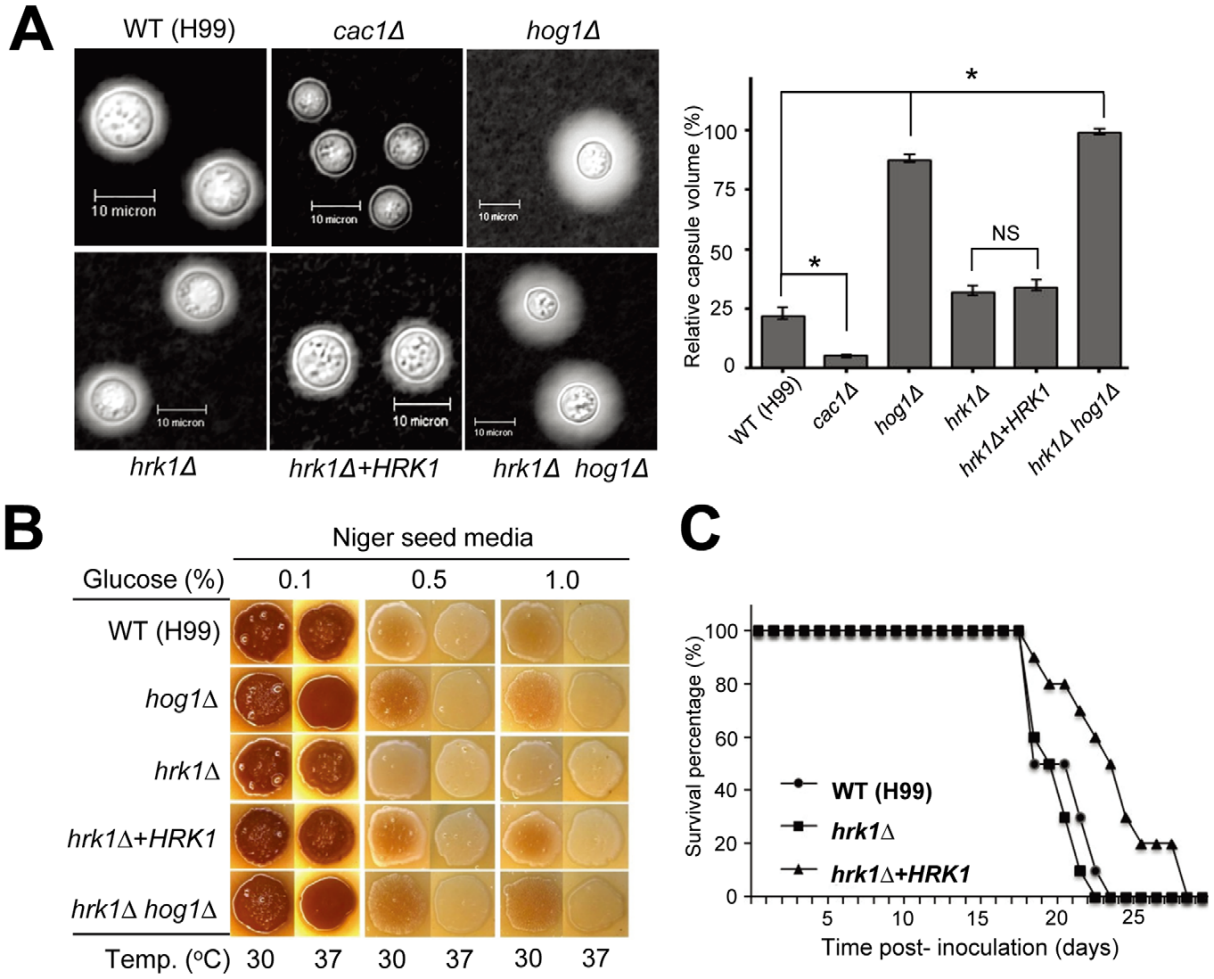
The role of Hrk1 in capsule and melanin production and virulence of *C. neoformans*. (A) Each *C. neoformans* strain were spotted and cultured on DME medium for capsule production at 37°C for 2 days. Capsule was visualized by staining with India ink and observed by microscopy (Bar, 10 µm). Quantitative measurements of the relative capsule volume (right panel). The packed volume of the cells was measured by calculating the ratio of the length of packed cell volume phase/length of total volume phase. Statistical differences in relative capsule size between strains were determined by Bonferroni's multiple comparison test. Error bars indicate the standard deviations. *, *P*<0.001. (B) Each strain was spotted and grown on Niger seed medium (glucose 0.1, 0.5, and 1%) at 30°C or 37°C for 5 days. (C) For virulence assay, groups of six- to eight-week old female A/J mice were infected with 5×10^4^ cells in 0.05 ml PBS by intranasal inhalation. Percent survival (%) was monitored daily until all mice were sacrificed. The *hrk1Δ* mutant is as virulent as the WT strain.

Another key feature of the *Cryptococcus* HOG pathway is its involvement in production of two major virulence factors, capsule and melanin, and sexual differentiation [5], [6], [8]. Perturbation of the HOG pathway increases capsule and melanin production in the *C. neoformans* H99 strain background. Our previous transcriptome analysis revealed that expression of the polysaccharide capsule biosynthesis genes, including *CAP10*, *CAP59*, *CAP60* and *CAP64*, is induced by perturbation of the HOG pathway [9]. Therefore, we have monitored capsule and melanin production in the *hrk1Δ* mutant. The *hrk1Δ* mutant displayed normal levels of capsule production comparable to the wild-type strain, whereas the *hog1Δ* mutant showed increased capsule production as previously described [5], [6], [8]. Furthermore the *hrk1Δ* mutation did not affect increased capsule production of the *hog1Δ* mutant (Fig. 7A). Therefore, Hrk1 does not appear to be involved in the Hog1-regulated capsule biosynthesis.

We have also monitored melanin biosynthesis levels in the *hrk1Δ* mutant. Previously we have reported that mutation of the HOG pathway increases *LAC1* expression and subsequently enhances melanin production [9]. Unlike the *hog1Δ* mutant, deletion of *HRK1* did not increase but instead slightly decreased melanin production (Fig. 7B), suggesting that Hrk1 plays a minor role in melanin biosynthesis.

The HOG pathway is negatively involved in sexual differentiation of *C. neoformans* by repressing pheromone production under normal conditions [5]. However, Hrk1 was not involved in sexual differentiation of *C. neoformans*. Even in a bilateral cross between *MAT*α and *MAT* **a** *hrk1Δ* mutants, normal filamentous growth and basidia and basidospore formation were observed similar to the cross between wild-type strains (Fig. S6). Thus, we conclude that Hrk1 is not involved in sexual differentiation of *C. neoformans*.

### Hrk1 is dispensable for virulence of *C. neoformans*

The role of Hrk1 in osmoadaptation, oxidative stress response, and melanin biosynthesis prompted us to investigate its role in virulence of *C. neoformans*, given the importance of these factors for survival within a host organism. Mice infected with the *hrk1Δ* mutant showed symptoms of sickness similar to those infected with the wild-type strain (Fig. 7C). The *hrk1Δ*+*HRK1* complemented strain exhibited slightly reduced virulence compared to the wild-type strain. During construction of the *hrk1Δ*+*HRK1* mutant, repeated biolistic transformation may affect its normal virulence (Fig. 7C). These results suggest that Hrk1 is dispensable for virulence of *C. neoformans*.

## Discussion

**Figure 8:**
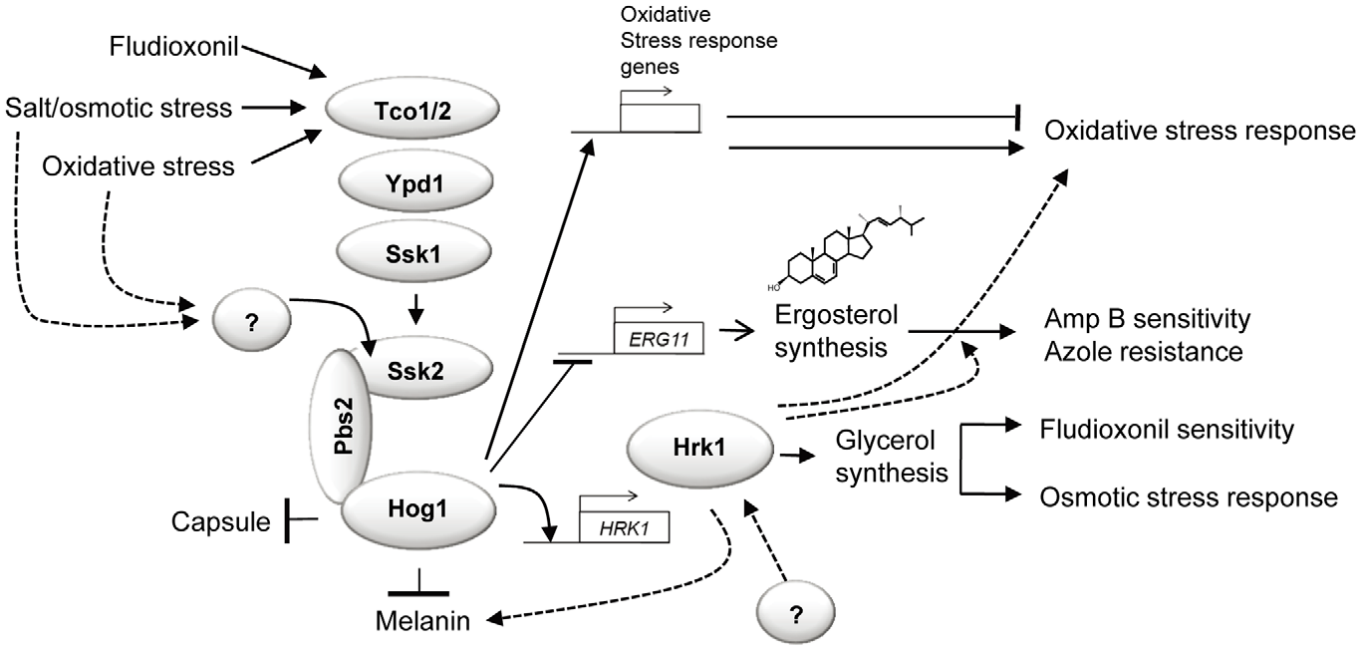
The proposed model for function and regulation of *C. neoformans* Hrk1. Basal and stress-induced expressions of *HRK1* are governed by the Hog1 MAPK. An unknown signaling component upstream of the Ssk2 MAPKKK, other than the Ssk1 response regulator, may trigger the Hog1 MAPK module (Ssk2-Pbs2-Hog1) in response to osmotic and oxidative stresses. Hrk1 is involved in intracellular glycerol synthesis upon fludioxonil treatment via the HOG pathway. However, Hrk1 appears to play several Hog1-independent roles. Independent of the HOG pathway modulating ergosterol biosynthesis, Hrk1 promotes susceptibility to polyene and azole drugs. Furthermore, Hrk1 is partly involved in promoting resistance to oxidative damaging agents, such as diamide, and melanin biosynthesis, which is in stark contrast to Hog1. In conclusion, Hrk1 plays both Hog1-dependent and –independent roles in *C. neoformans*.

In this study we characterized functions of a novel Hog1-regulated kinase, Hrk1, in *C. neoformans* by molecular and genetic analyses. Hrk1, identified by our prior transcriptome analysis of the HOG pathway, was confirmed to be differentially regulated via the Hog1 MAPK in response to environmental stresses, such as osmotic shock and oxidative stress. Hog1 is required for the high basal levels of Hrk1, probably due to high Hog1-phosphorylation levels under basal conditions. Similar to the HOG mutants, the *hrk1Δ* mutants showed increased resistance to fludioxonil by inhibiting over-accumulation of intracellular glycerol and cell swelling. However, Hrk1 appears to have a number of Hog1-independent functions. Hrk1 played a minor role in maintaining cellular osmotic balance but in a Hog1-independent manner because deletion of *HRK1* exacerbated osmosensitivity of the *hog1Δ* mutant. Furthermore, the *hrk1Δ* mutant showed distinct oxidative stress response patterns from the *hog1Δ* mutant. The *hrk1Δ* mutation increases diamide sensitivity of the wild-type and further enhances H_2_O_2_ sensitivity of the *hog1Δ* mutant. Finally, *hrk1Δ* mutation suppresses azole resistance shown by the *hog1Δ* mutant without affecting *ERG11* expression. Taken together, Hrk1 not only mediates a subset of HOG-related phenotypes, but also appears to be implicated in several HOG-independent phenotypes (see the proposed model for the role of Hrk1 in *C. neoformans* in Fig. 8).

Hrk1 is structurally close to other p38/Hog1-dependent MAPKAPKs, including Rck1 and Rck2 in *S. cerevisiae*, and Srk1/Mkp1 and Cmk2/Mkp2 in *S. pombe*. Although Hrk1 is most homologous to Srk1, the closest *C. neoformans* homolog to Cmk2, Rck1, and Rck2 is also Hrk1, indicating that Hrk1 may share functions with either of the MAPKAPKs. Furthermore, none of other *C. neoformans* genes display any significant sequence homology to the Srk1- or Rck2-like MAPKAPKs. Phylogenetic analysis of conserved gene families in the fission and budding yeasts revealed that Rck2 and Srk1 share a common ancestor but Rck1-Rck2 and Srk1-Cmk2 appear to be independently duplicated in *S. cerevisiae* and *S. pombe*, respectively [22]. Therefore, Hrk1 is likely to share the common ancestor with Srk1 and Rck2 but is not duplicated in *C. neoformans*. Although the MAPKAPKs exhibit high similarity to Ca^2+^/calmodulin-dependent kinases, none of them is involved in Ca^2+^-signaling. Srk1 has been identified by microarray analysis to be one of only two protein kinase genes induced by stress in the fission yeast [23]. Srk1 is transcriptionally upregulated in response to a range of stressors, including H_2_O_2_, osmotic and heat shock, and its induction is completely abolished in the *sty1Δ* mutant [11]. Rck1 and Rck2 in the budding yeast were initially discovered as suppressors of fission yeast checkpoint mutation (radiation and hydroxyurea sensitivity) [24]. Later Rck2 was also independently identified as a Hog1-interacting protein via yeast-two hybrid screen and found to be the direct phosphorylation target of Hog1 in response to osmotic shock [14], [15]. Similar to *SRK1*, *HRK1* expression is almost abolished in the *hog1Δ* mutant. Conversely, *HRK1* expression patterns appear to be different from *SRK1* in response to environmental signals. *HRK1* expression is downregulated by osmotic stress, but upregulated by oxidative stress (Fig. 2). Another difference between Hrk1 and Srk1 is its basal expression levels. Basal expression levels of *SRK1* appear to be minimal under unstressed conditions [11] whereas basal expression levels of *HRK1* are high even under unstressed conditions (Figs. 1B and 2). This discrepancy may result from the fact that *C. neoformans* Hog1 is constitutively phosphorylated under unstressed conditions whereas *S. pombe* Sty1 is not.

Basal expression levels of *HRK1* are mainly governed by the Ssk1 response regulator and Hog1 MAPK. Interestingly, though, *HRK1* can be strongly induced in response to oxidative and osmotic stress in the *ssk1Δ* mutant background, strongly indicating that unknown signaling component(s), other than Ssk1, can activate Hog1 and subsequently induce expression of Hrk1. In response to osmotic stress (1 M NaCl), *HRK1* expression is slowly recovered to the wild-type levels in the *ssk1* mutant although it is not known whether the same signaling component(s) is involved in both the oxidative and osmotic stress response. These findings correspond to our previous observation that constitutive phosphorylation of Hog1 almost disappears in the *ssk1Δ* mutant but its phosphorylation can be induced by 1 M NaCl [6]. This signaling component(s) should exist upstream of the Ssk2 MAPKKK and Pbs2 MAPKK since Hog1 phosphorylation is not induced in the *ssk2Δ* or *pbs2Δ* mutant [5], [8]. The identity of the Ssk1-independent signaling component(s) upstream of the Ssk2-Pbs2-Hog1 MAPK module will be elucidated in future studies.

In addition to different expression patterns, functions of Hrk1 appear to be distinct from those of other Hrk1 orthologs, such as Srk1, Cmk2, Rck1 or Rck2. Although Srk1 is directly phosphorylated by Sty1 and translocated into the nucleus in *S. pombe*, the *srk1Δ* mutant does not exhibit any discernable stress-related phenotypes. Instead, Srk1 was found to repress meiosis and sporulation in the fission yeast whereas Cmk2/Mkp2 is not involved in the mating and meiotic processes [11], [20]. Cmk2 is involved in oxidative stress response (against H_2_O_2_ and sodium arsenite) in *S. pombe* [10]. Similarly, although Rck2 is phosphorylated by Hog1 in response to osmotic stress in *S. cerevisiae* and overexpression of *RCK2* restores salt resistance of the *hog1Δ* mutant, deletion of either *RCK2* or *RCK1*, or both does not cause any obvious osmosensitive phenotypes and does not influence activity of glycerol-3-phosphate dehydrogenase and catalase [14], [15]. Furthermore, the *rck1Δ*, *rck2Δ*, or *rck1Δ rck2Δ* mutants do not show any other abnormalities in growth, morphology, conjugation, and actin cytoskeleton regulation [24]. Similar to Srk1, however, both Rck1 and Rck2 repress meiosis and sporulation [12]. Rck1 and Rck2 are also involved in oxidative stress response [16], similar to *S. pombe* Cmk2. The *rck2Δ* mutant is more sensitive to tBOOH and cadmium than WT whereas the *rck1Δ* mutant is only slightly more sensitive to tBOOH [16]. In contrast, Hrk1 plays a substantial role in response and adaptation to diverse environmental stresses and antifungal drugs. Hrk1 is required for intracellular glycerol synthesis via the HOG pathway and thereby controls sensitivity to the antifungal drug fludioxonil (Fig. 4). Hrk1 is required for resistance to both osmotic stress and H_2_O_2_ in the absence of the Hog1 MAPK. In addition, Hrk1 mediates resistance to diamide in a Hog1-independent manner (Fig. 4 and 5). In conclusion, Hrk1 plays more significant roles in environmental stress response than Srk1 and Cmk2 in *S. pombe* or Rck1 and Rck2 in *S. cerevisiae*.

Regardless of the stress-related and virulence factor-related phenotypes, Hrk1 was found to be dispensable for virulence of *C. neoformans*, suggesting that single antifungal therapy with any Hrk1 inhibitors will not be effective for treatment of cryptococcosis. Nevertheless, Hrk1 could be a good target for combination antifungal therapy with azole drugs. Inhibition of Hrk1 increases fluconazole-sensitivity of *C. neoformans*. Notably, mutation of *HRK1* completely suppresses azole-resistance of the *hog1Δ* mutant. Indeed the *hrk1Δ hog1Δ* double mutant displayed increased susceptibility to both fluconazole and ketoconazole. Since the *hrk1Δ* mutant exhibits nearly wild-type levels of amphotericin B resistance, Hrk1 does not appear to be directly involved in ergosterol biosynthesis. Supporting this, *ERG11* expression was not altered by deletion of *HRK1*. These results indicate that simultaneous inhibition of the HOG pathway and Hrk1 along with combination of either polyene or azole drugs could be a good combination antifungal therapy for treatment of cryptococcosis. In future studies, it needs to be further investigated how Hrk1 promotes azole-resistance in *C. neoformans*.

In conclusion, Hrk1 plays both redundant and distinct roles with the HOG pathway in environmental stress and antifungal drug response and virulence factor production in *C. neoformans*. Hrk1 is a protein kinase downstream of the Hog1 MAPK and yet has the potential to be modulated by other signaling pathways, which will be elucidated in future studies.

## Materials and Methods

### Ethics Statement

All animal experiments were done at the University of Minnesota in strict accordance with good animal practice as defined by the National Institutes of Health Office of Animal Welfare (OLAW), and the Association for the Assessment and Accreditation of Laboratory Animal Care (AAALAC). All experiments were reviewed and approved by the University of Minnesota Institutional Animal Care and Use Committee (IACUC) under protocol number 0712A22250.

### Strains and growth conditions

*C. neoformans* strains used in this study are listed in Table 1 and were cultured in YPD (yeast extract-peptone-dextrose) medium. Agar-based DME (Dulbecco's modified Eagle's) medium (Invitrogen, Carlsbad, CA) for capsule production, Niger seed or L-DOPA medium for melanin production, and V8 mating medium were prepared as previously described [25]. For stress and antifungal drug sensitivity, cells were grown as follows. Each strain was incubated overnight at 30°C in YPD medium, washed, serially diluted (1 to 10^4^ dilutions) in dH_2_O, and spotted (4 µl) onto solid YPD medium. To test osmosensitivity, cells were spotted on solid YP (no glucose) or YPD medium containing 1 M or 1.5 M of NaCl or KCl. To test sensitivity to antifungal drugs, cells were spotted on solid YPD medium containing the indicated concentration of fludioxonil (PESTANAL®; Sigma), fluconazole (Sigma), ketoconazole (Sigma), or amphotericin B (Sigma). To examine oxidative stress, the cells were spotted on YPD containing the indicated concentration of H_2_O_2_ (Junsei), diamide, *tert*-butyl hydroperoxide (tBOOH), or menadione. To test sensitivity to UV, cells spotted on solid YPD medium were exposed to UV irradiation for 0.2 (480 J/m^2^) or 0.3 (720 J/m^2^) min using a UV Stratalinker™ (Model 2400, Stratagene). To test temperature sensitivity, plates were incubated at 30, 37, and 40°C. Each plate was incubated for 2–5 days, and photographed.

**Table 1:** *C. neoformans* strains used in this study.

Strain	Genotype	Parent	Reference
**Serotype A**			
H99	*MAT*α		[30]
KN99**a**	*MAT* **a**		[31]
YSB42	*MAT*α *cac1Δ::NAT-STM#177*	H99	[25]
YSB64	*MAT*α *hog1Δ::NAT-STM#177*	H99	[5]
YSB81	*MAT* **a** *hog1Δ::NEO*	KN99**a**	[5]
YSB261	*MAT*α *ssk1Δ::NAT-STM#205*	H99	[6]
YSB349	*MAT*α *skn7Δ::NAT-STM#201*	H99	[6]
YSB270	*MAT*α *hrk1Δ::NAT-STM#58*	H99	This study
YSB874	*MAT* **a** *hrk1Δ::NEO*	KN99**a**	This study
YSB883	*MAT*α *hrk1Δ::NAT-STM#58 HRK1-NEO*	YSB270	This study
YSB988	*MAT*α *hrk1Δ::NAT-STM#58 hog1Δ::NEO*	YSB270	This study

Each *NAT-STM#* indicates the Nat^r^ marker with a unique signature tag.

### Quantitative reverse transcriptase-PCR (qRT-PCR) analysis

The qRT-PCR for quantitatively measuring relative expression levels of *HRK1* was performed with primers listed in Supplementary Table S1. cDNAs were synthesized using the SuperScript II reverse transcriptase system with three independent sets of total RNA samples that were prepared for the previous DNA microarray analysis of the HOG pathway in *C. neoformans* [9]. Relative gene expression of *HRK1* was calculated by the threshold cycle 2^−ΔΔCT^ method [26] from three biological replicates and three technical replicates. Transcript levels of the *ACT1* gene were used for normalization.

### Disruption of the *HRK* (Hog1-regulated kinase) gene

To characterize the function of Hrk1, the *HRK1* gene was deleted in the genetic background of *C. neoformans* serotype A strain H99 (*MAT*α) and KN99**a** (*MAT* **a**) with a disruption cassette generated by overlap PCR and by biolistic transformation as previously described [5], [27]. Primers for amplification of the 5′ and 3′ flanking regions of the *HRK1* gene and Nat^r^ selectable markers are described in supplemental Table S1. Stable transformants were selected on YPD medium containing nourseothricin. The *hrk1Δ* mutant strain was confirmed by both diagnostic PCR and by Southern blot analysis using a gene-specific probe prepared by primers listed in supplemental Table S1 (Fig. S2). To construct the *hrk1Δ hog1Δ* double mutant, the *hog1Δ* mutant (YSB64) was biolistically transformed with the *hrk1Δ* disruption cassettes containing a Neo^r^ selectable marker and confirmed by diagnostic PCR and Southern blot analysis (Fig. S3).

To authenticate *hrk1* mutant phenotypes, the *hrk1Δ*+*HRK1* complemented strains were constructed as follows. First, the 4.2-kb fragment containing the complete ORF of the *HRK1* gene was amplified by PCR with primers B32 and B33 (Table S1) and cloned into the pCR2.1-TOPO vector (Invitrogen), generating pCR-HRK1. After confirming the DNA sequence, the *HRK1* insert was subcloned into plasmid pJAF12 (NEO^r^), generating plasmid pNEOHRK1. For the targeted re-integration of the wild-type *HRK1* allele into its native locus, pNEOHRK1 was linearized by BbsI digestion and biolistically transformed into the *hrk1* strain YSB270 (Table 1).

### Southern, Northern, and Western blot analysis

For Southern blot analysis, genomic DNAs were isolated from each strain using CTAB (Cetyl trimethylammonium bromide, Sigma) extraction buffer as described previously [28]. Isolated genomic DNA was digested with the indicated restriction enzymes (i.e. XbaI and XhoI for checking deletion of the *HRK1* gene and BamHI for checking the *hrk1Δhog1Δ* double mutant). The digested genomic DNAs were separated by 1% agarose gel-electrophoresis, denatured in 0.4 N NaOH, transferred to the nylon membrane (GE) in 0.4 N NaOH and 1 M NaCl, and fixed by 1200 J/m^2^ UV exposure. The membrane was hybridized, washed, and developed as described before [28]. For Northern blot analysis, each strain was grown to mid-logarithmic phase in YPD medium at 30°C. Total RNAs were isolated using Trizol (Invitrogen) or Ribo-EX (Geneall) as described before [28]. Northern blot analysis was performed with 10 µg of total RNA from each strain. Electrophoresis, membrane transfer, hybridization, and washing as described before [28]. Gene specific probes were prepared by PCR amplification with gene specific primers listed in Table S1. Western blot analysis for monitoring Hog1 phosphorylation was performed as previously described [5]. To detect phosphorylated *C. neoformans* Hog1, rabbit p38-MAPK specific antibody (Cell Signaling) and anti-rabbit IgG horseradish peroxidase-conjugated antibody were used as primary and secondary antibodies, respectively. The blot was developed using the ECL Western Blotting Detection System (Amersham Bioscience). Subsequently, the blot was stripped and used for detection of Hog1 with a rabbit polyclonal anti-Hog1 antibody (Santa Cruz Biotechnology) as a loading control.

### Assays for capsule and melanin production and mating

Qualitative visualization of capsule and melanin production was performed as described previously [25], [29]. Mating assay was performed as described previously [25], [29].

### Intracellular glycerol measurement

Each strain was grown to mid-logarithmic phase. Cells were subcultured into YPD liquid medium with or without fludioxonil (final 10 µg/ml) for 48 hrs at 30°C with shaking. After incubation, cell morphologies were observed by microscope and photographed. To measure the intracellular glycerol content, cells were collected by centrifugation, washed with distilled water and then extracted by boiling at 100°C for 10 minutes. After centrifugation, the glycerol concentration in the supernatant was measured using a Glycerol UV-method kit according to the manuals (Roche). The same amount of each sample was lyophilized to measure cellular dry weight for normalization.

### Virulence assay

For animal infections, overnight cultures of *C. neoformans* strains H99, *hrk1Δ* (YSB270), *hrk1Δ*+*HRK1* (YSB568) were grown in YPD broth at 30°C. The resulting cultured yeast cells were pelleted and washed 3 times in sterile PBS and concentrations were adjusted to 1×10^6^ cells/ml for each strain based on hemocytometer count. Virulence studies were performed using the murine inhalation model. Groups of six- to eight-week old female A/J mice (Jackson Labs, Bar Harbor, MA) were anesthetized by intraperitoneal pentobarbital injection. Mice were infected with 5×10^4^ cells in 0.05 ml PBS by intranasal inhalation. Initial concentrations were confirmed by plating serial dilutions on YPD agar and enumerating colony-forming units (CFUs). Mice were monitored daily and those showing signs of severe morbidity (weight loss, abnormal gait, extension of the cerebral portion of the cranium) were sacrificed by CO_2_ inhalation. Homogenized lung tissue was plated onto YPD agar or YPD media containing nourseothricin (200 mg/ml) or neomycin (100 mg/ml) from representative animals from each infection to verify the genotype of the recovered cryptococcal strains. All animal experiments were reviewed and approved by the University of Minnesota Institutional Animal Care and Use Committee (IACUC) under protocol number 0712A22250. Survival data from the mouse experiment were analyzed by Analyse-It for Excel.

## Supporting Information

Figure S1 **Northern blot analysis of stress-dependent expression patterns of** ***HRK1*** **in** ***C. neoformans*** **.** Total RNA was isolated from WT and the *ssk1Δ* and *hog1Δ* mutants grown in YPD medium containing 1 M NaCl for osmotic stress (A), 2.5 mM H_2_O_2_ for oxidative stress (B), or 40 µg/ml fludioxonil for antifungal drug treatment (C) at different time points (0, 30 and 60 min).
(PPT)

Figure S2 **Construction of the serotype A** ***MAT*** **α** ***hrk1Δ*** **mutant.** (A) Diagram for disruption of the *HRK1* gene in serotype A *MAT*α strain H99. Primers for the first-round and second-round PCR are indicated as bent arrows. Through recombination between 5′ and 3′ flanking region of the *HRK1* gene, the intact *HRK1* gene is replaced with nourseothricin-resistant gene (*NAT*). (B) The correct genotype of the *hrk1*Δ mutants was confirmed by Southern blot analysis using genomic DNA digested with the restriction enzyme XhoI and XbaI.
(PPT)

Figure S3 **Construction of the serotype A** ***MAT*** **α** ***hrk1Δ hog1Δ*** **double mutant.** (A) Diagram for disruption of the *HOG1* gene in YSB270 (*hrk1Δ* α) strain. Primers for the first-round and second-round PCR are indicated as bent arrows. Through recombination between 5′ and 3′ flanking region of *HOG* gene, the intact *HOG1* gene is displaced with neomycin-resistant gene (*NEO*). (B) The correct genotype of the *hrk1Δ hog1Δ* double mutants was confirmed by Southern blot analysis using genomic DNA digested with the restriction enzyme BamHΙ.
(PPT)

Figure S4 **Western blot analysis of Hog1 phosphorylation in WT and** ***hrk1Δ*** **mutant.** The WT (strain H99) and *hrk1Δ* (YSB270) mutant strains were grown to the mid-logarithmic phase and exposed to 1 M NaCl in YPD medium for the indicated amount of time and total protein extracts were prepared. The dual phosphorylation status of Hog1 (T171 and Y173) was examined by using anti-dually phosphorylated p38 antibody (Hog1-P). Subsequently the same blots were stripped and reprobed with polyclonal anti-Hog1 antibody as a loading control (Hog1).
(PPT)

Figure S5 **Hrk1 does not play a role in resistance to high temperature, UV irradiation, and cell membrane/wall integrity destabilizers.** Each *C. neoformans* strain indicated below was grown overnight (about 16 hrs) at 30°C in liquid YPD medium, 10-fold serially diluted (1–10^4^ dilutions), and spotted (4 µl of dilution) on YPD agar containing the indicated concentrations of SDS, DTT, Congo red, and H_2_O_2_. To test genotoxic DNA damaging stress, cells were spotted on solid YPD medium and exposed to 400 J/m^2^ of UV by using a UV crosslinker (UVP). To test temperature sensitivity, plates were incubated at 38°C for 3 days. The wild-type H99, *ssk1*Δ (YSB261), *hog1Δ* (YSB64), *skn7Δ* (YSB349), *hrk1Δ* (YSB270), *hrk1Δ*+*HRK1* (YSB883), and *hrk1Δ hog1Δ* (YSB988)] strains were used.
(PPT)

Figure S6 **Hrk1 is not required for sexual differentiation in serotype A** ***C. neoformans*** **strain.** Each *MAT*α and *MAT*a strains were co-incubated on V8 medium (pH 5.0) for up to 15 days at room temperature in the dark: WT α×WT **a** (H99 and KN99**a**), *hog1Δ* α×*hog1Δ* **a** (YSB64 and YSB81), *hrk1Δ* α×*hrk1Δ* **a** (YSB270 and YSB874). The images were photographed after 7 and 15 days.
(PPT)

Table S1 **Oligonucleotide primers used in this study.**
(DOC)
