# Supplementary material for: Hrk1 Plays Both Hog1-Dependent and -Independent Roles in Controlling Stress Response and Antifungal Drug Resistance in Cryptococcus neoformans
Source: PLoS One. 2011 Apr 13;6(4):e18769. doi: 10.1371/journal.pone.0018769 (PMC3076434; doi:10.1371/journal.pone.0018769)
Supplement: Figure S1 — Northern blot analysis of stress-dependent expression patterns of HRK1 in C. neoformans . Total RNA was isolated from WT and the ssk1Δ and hog1Δ mutants grown in YPD medium containing 1 M NaCl for osmotic stress (A), 2.5 mM H2O2 for oxidative stress (B), or 40 µg/ml fludioxonil for antifungal drug treatment (C) at different time points (0, 30 and 60 min). (PPT) [file pone.0018769.s001.ppt]

## Slide 1
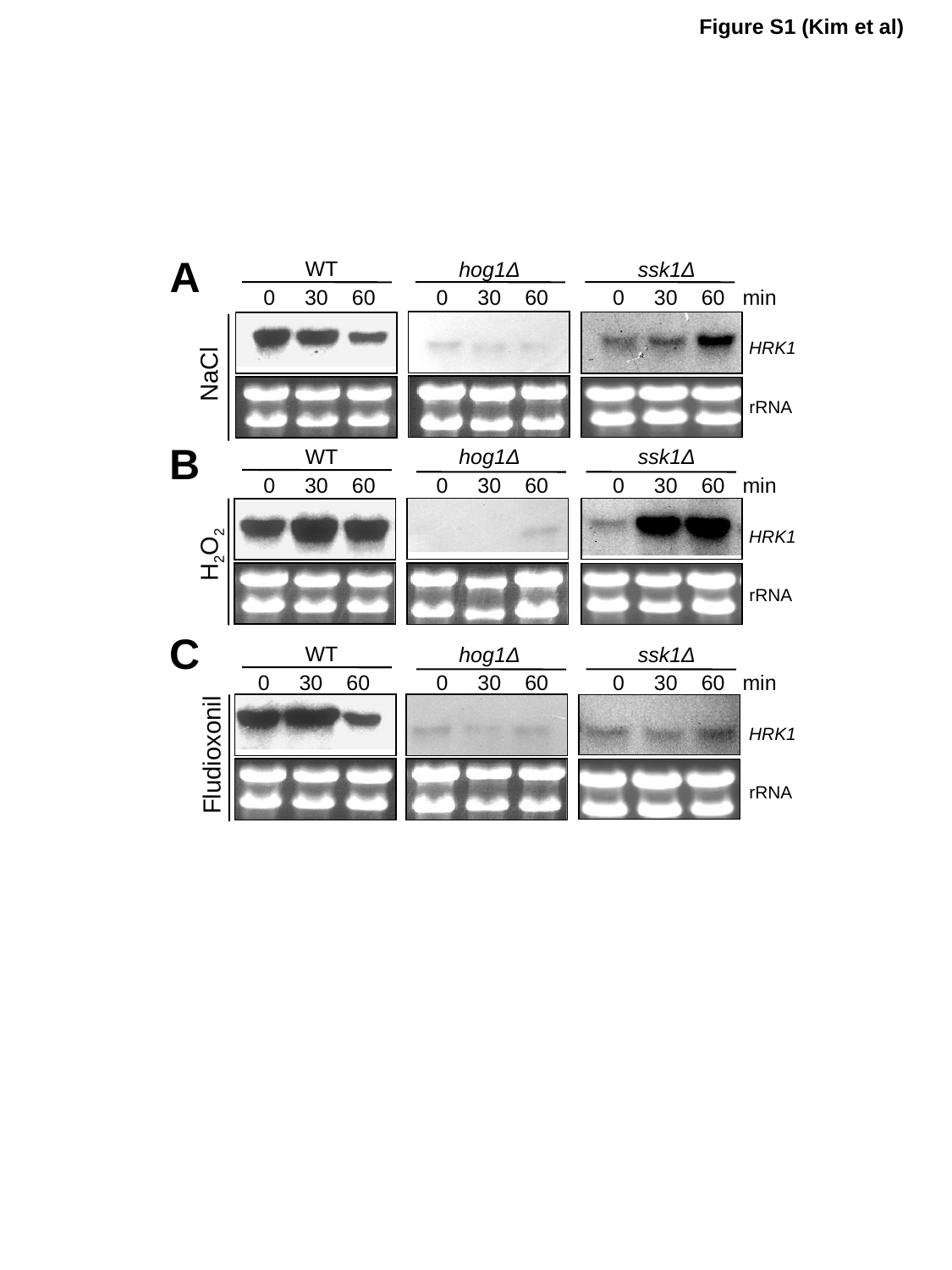

Figure S1 (Kim et al)
A
WT
hog1Δ
ssk1Δ
0 30 60
0 30 60
0 30 60 min
HRK1
rRNA
NaCl
B
WT
hog1Δ
ssk1Δ
0 30 60
0 30 60
0 30 60 min
HRK1
rRNA
H2O2
C
WT
hog1Δ
ssk1Δ
0 30 60
0 30 60
0 30 60 min
HRK1
rRNA
Fludioxonil
