# Supplementary figures and images for: Hrk1 Plays Both Hog1-Dependent and -Independent Roles in Controlling Stress Response and Antifungal Drug Resistance in Cryptococcus neoformans
Source: PLoS One. 2011 Apr 13;6(4):e18769. doi: 10.1371/journal.pone.0018769 (PMC3076434; doi:10.1371/journal.pone.0018769)

## Slide 1
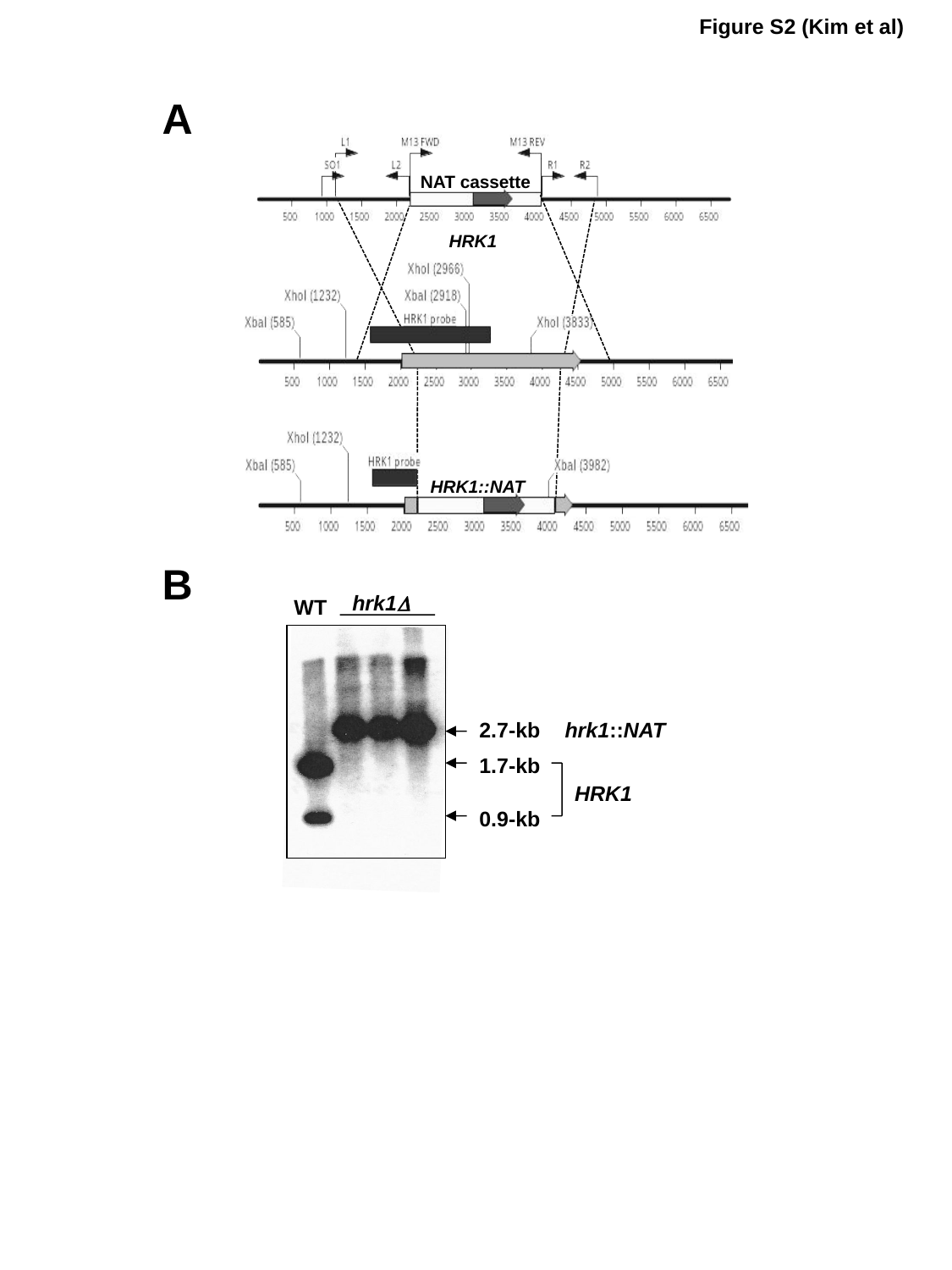

Figure S2 (Kim et al)
A
NAT cassette
HRK1
HRK1::NAT
B
hrk1Δ
WT
2.7-kb
hrk1::NAT
1.7-kb
HRK1
0.9-kb

Supplement: Figure S2 — Construction of the serotype A MAT α hrk1Δ mutant. (A) Diagram for disruption of the HRK1 gene in serotype A MATα strain H99. Primers for the first-round and second-round PCR are indicated as bent arrows. Through recombination between 5′ and 3′ flanking region of the HRK1 gene, the intact HRK1 gene is replaced with nourseothricin-resistant gene (NAT). (B) The correct genotype of the hrk1Δ mutants was confirmed by Southern blot analysis using genomic DNA digested with the restriction enzyme XhoI and XbaI. (PPT) [file pone.0018769.s002.ppt]

## Slide 1
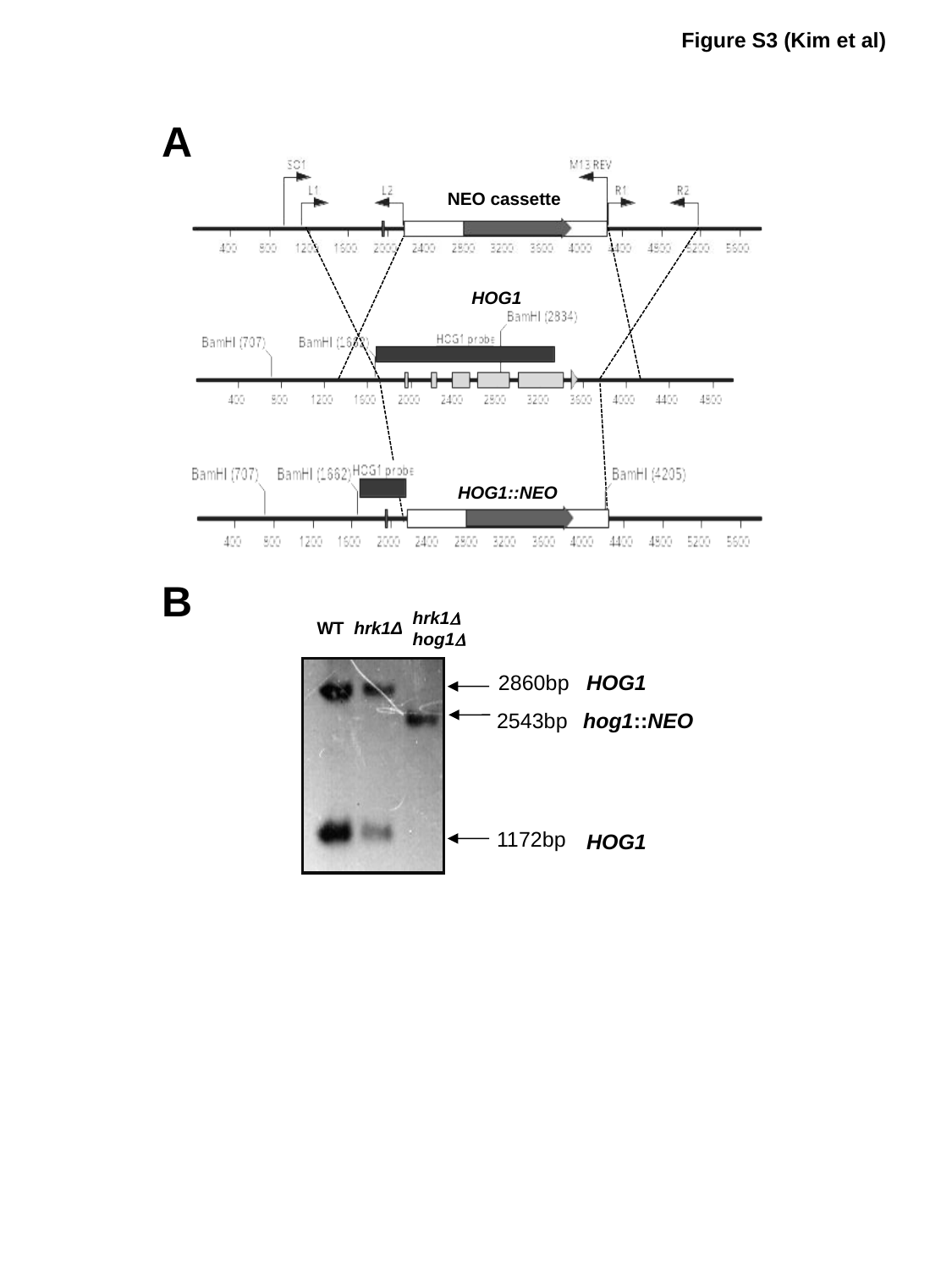

Figure S3 (Kim et al)
A
NEO cassette
HOG1
HOG1::NEO
B
hrk1Δ
hog1Δ
WT hrk1Δ
2860bp
HOG1
2543bp
hog1::NEO
1172bp
HOG1

Supplement: Figure S3 — Construction of the serotype A MAT α hrk1Δ hog1Δ double mutant. (A) Diagram for disruption of the HOG1 gene in YSB270 (hrk1Δ α) strain. Primers for the first-round and second-round PCR are indicated as bent arrows. Through recombination between 5′ and 3′ flanking region of HOG gene, the intact HOG1 gene is displaced with neomycin-resistant gene (NEO). (B) The correct genotype of the hrk1Δ hog1Δ double mutants was confirmed by Southern blot analysis using genomic DNA digested with the restriction enzyme BamHΙ. (PPT) [file pone.0018769.s003.ppt]

## Slide 1
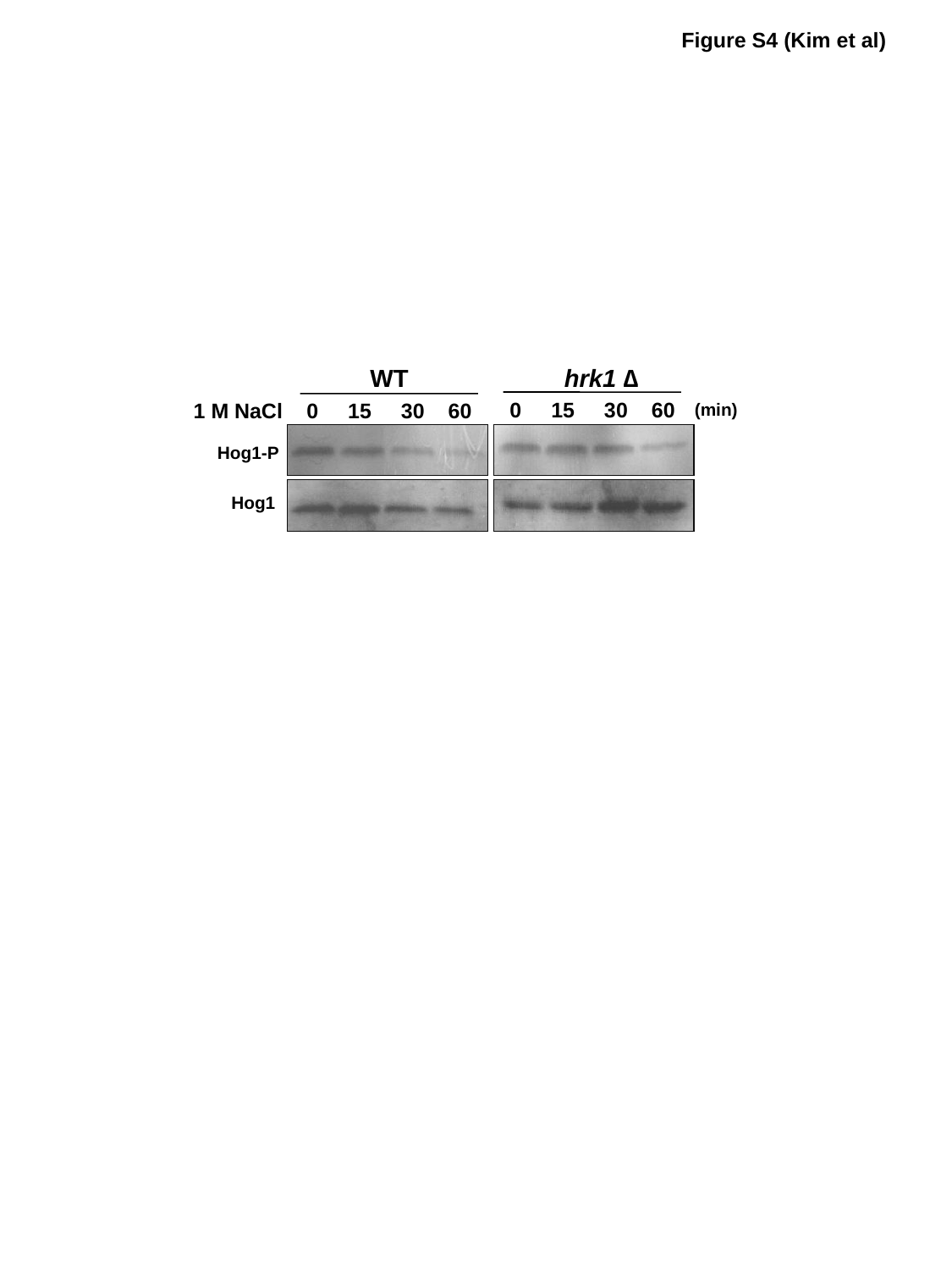

Figure S4 (Kim et al)
WT
hrk1 ∆
0 15 30 60
1 M NaCl
0 15 30 60
(min)
Hog1-P
Hog1

Supplement: Figure S4 — Western blot analysis of Hog1 phosphorylation in WT and hrk1Δ mutant. The WT (strain H99) and hrk1Δ (YSB270) mutant strains were grown to the mid-logarithmic phase and exposed to 1 M NaCl in YPD medium for the indicated amount of time and total protein extracts were prepared. The dual phosphorylation status of Hog1 (T171 and Y173) was examined by using anti-dually phosphorylated p38 antibody (Hog1-P). Subsequently the same blots were stripped and reprobed with polyclonal anti-Hog1 antibody as a loading control (Hog1). (PPT) [file pone.0018769.s004.ppt]

## Slide 1
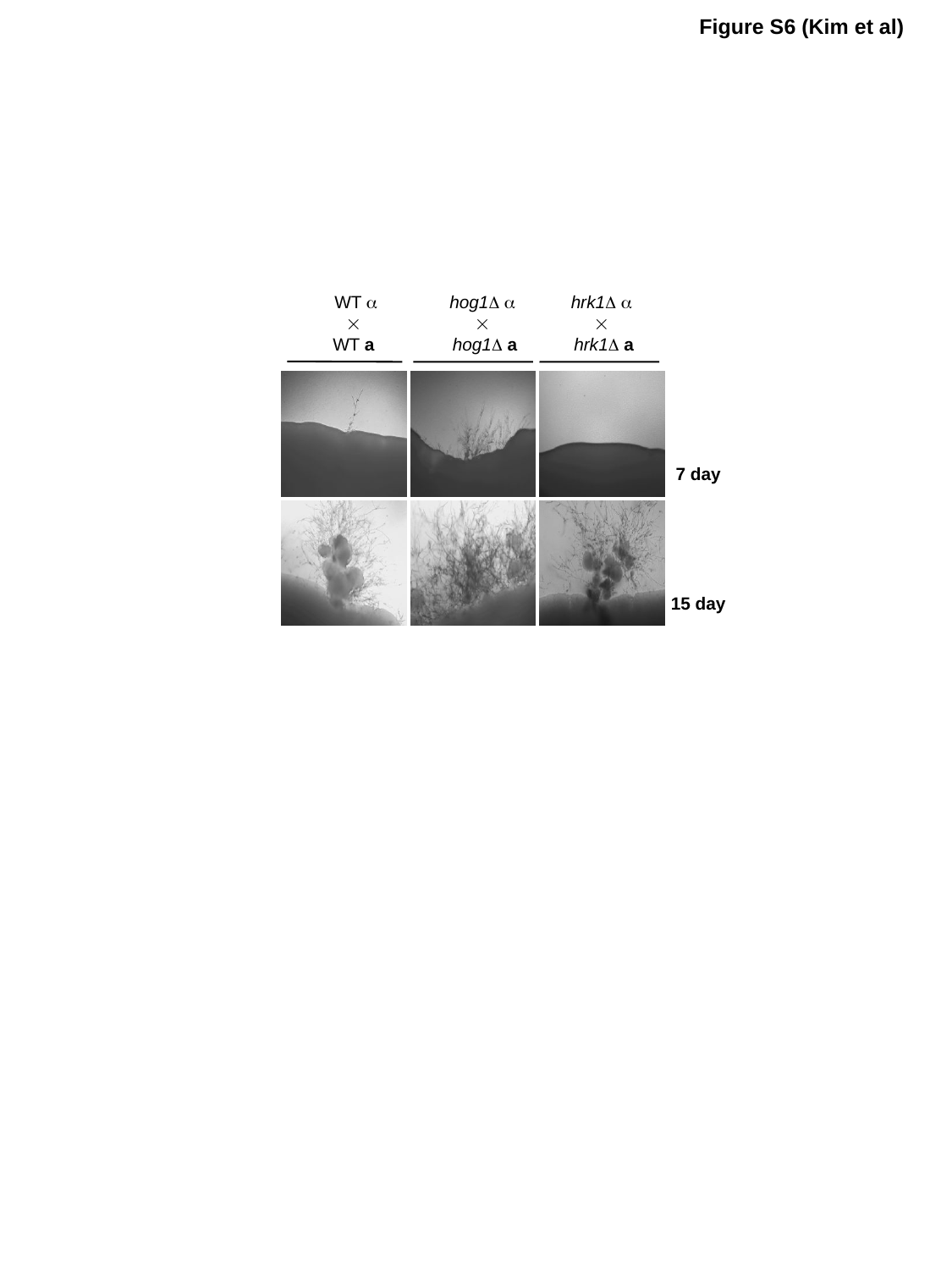

Figure S6 (Kim et al)
WT α
×
WT a
hog1Δ α
×
hog1Δ a
hrk1Δ α
×
hrk1Δ a
7 day
15 day

Supplement: Figure S6 — Hrk1 is not required for sexual differentiation in serotype A C. neoformans strain. Each MATα and MATa strains were co-incubated on V8 medium (pH 5.0) for up to 15 days at room temperature in the dark: WT α×WT a (H99 and KN99a), hog1Δ α×hog1Δ a (YSB64 and YSB81), hrk1Δ α×hrk1Δ a (YSB270 and YSB874). The images were photographed after 7 and 15 days. (PPT) [file pone.0018769.s006.ppt]
