# Supplementary material for: Hrk1 Plays Both Hog1-Dependent and -Independent Roles in Controlling Stress Response and Antifungal Drug Resistance in Cryptococcus neoformans
Source: PLoS One. 2011 Apr 13;6(4):e18769. doi: 10.1371/journal.pone.0018769 (PMC3076434; doi:10.1371/journal.pone.0018769)
Supplement: Figure S5 — Hrk1 does not play a role in resistance to high temperature, UV irradiation, and cell membrane/wall integrity destabilizers. Each C. neoformans strain indicated below was grown overnight (about 16 hrs) at 30°C in liquid YPD medium, 10-fold serially diluted (1–104 dilutions), and spotted (4 µl of dilution) on YPD agar containing the indicated concentrations of SDS, DTT, Congo red, and H2O2. To test genotoxic DNA damaging stress, cells were spotted on solid YPD medium and exposed to 400 J/m2 of UV by using a UV crosslinker (UVP). To test temperature sensitivity, plates were incubated at 38°C for 3 days. The wild-type H99, ssk1Δ (YSB261), hog1Δ (YSB64), skn7Δ (YSB349), hrk1Δ (YSB270), hrk1Δ+HRK1 (YSB883), and hrk1Δ hog1Δ (YSB988)] strains were used. (PPT) [file pone.0018769.s005.ppt]

## Slide 1
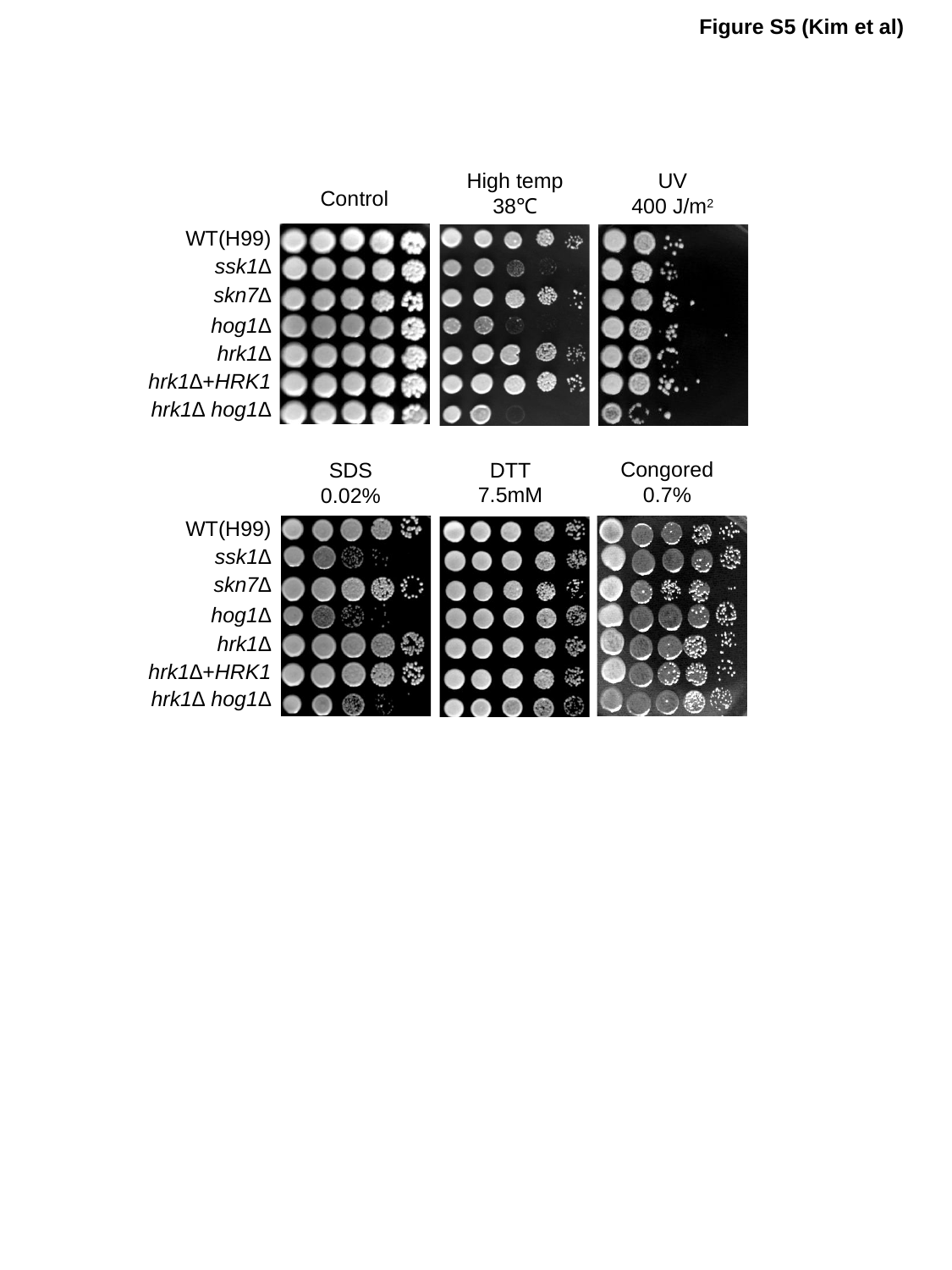

Figure S5 (Kim et al)
High temp
38℃
UV
400 J/m2
Control
WT(H99)
ssk1∆
skn7∆
hog1∆
hrk1∆
hrk1∆+HRK1
hrk1∆ hog1∆
Congored
0.7%
DTT
7.5mM
SDS
0.02%
WT(H99)
ssk1∆
skn7∆
hog1∆
hrk1∆
hrk1∆+HRK1
hrk1∆ hog1∆
