# Supplementary material for: Hrk1 Plays Both Hog1-Dependent and -Independent Roles in Controlling Stress Response and Antifungal Drug Resistance in Cryptococcus neoformans
Source: PLoS One. 2011 Apr 13;6(4):e18769. doi: 10.1371/journal.pone.0018769 (PMC3076434; doi:10.1371/journal.pone.0018769)
Supplement: Table S1 — Oligonucleotide primers used in this study. (DOC) [file pone.0018769.s007.doc]

**Table S1. Oligonucleotide primers used in this study**

| Primer | Sequence (5’-3’) | Comments |
| --- | --- | --- |
| B79 | TGTGGATGCTGGCGGAGGATA | Screening primer on ACT promter |
| Bahn1026 | GTAAAACGACGGCCAGTGAGC | M13 forward |
| Bahn1027 | CAGGAAACAGCTATGACCATG | M13 reverse |
| JOHE13990 | AAGACGACCACATCTCAGAG | Hrk1 in H99- 5’ screening primer |
| JOHE13986 | TTCCAGTCAACCGAGTAGC | Distruption construt for Hrk1 in H99- left flanking primer 1 |
| JOHE13987 | CTGGCCGTCGTTTTACCTGTATTCATCATTGCGGC | Distruption construt for Hrk1 in H99 - left flanking primer 2 |
| JOHE13988 | GTCATAGCTGTTTCCTGCGTCAAATCCAAGAACATCGTG | Distruption construt for Hrk1 in H99 - right flanking primer 1 |
| JOHE13989 | GCCTTCATCGTCGTTAGAC | Distruption construt for Hrk1 in H99 - right flanking primer 2 |
| JOHE13991 | AGGACTCTGCTCCATCAAG | Confirm Southern blot for *hrk1Δ* in KN99 |
| JOHE13992 | GAAAGAGCCTCAGAAAAGTAGG | Confirm Southern blot for *hrk1Δ* in KN99 |
| JOHE11799 | TCACAGAGCGTTGATTACG | Confirm Southern blot for *hrk1Δ hog1Δ* double mutant in H99 |
| JOHE11800 | AATCAAACACCTCGGCGGCAAC | Confirm Southern blot for *hrk1Δ hog1Δ* double mutant in H99 |
| Bahn32 | CGCGCGGCCGCAAGACGACCACATCTCAGAG | HRK1 in H99-left flanking primer (NotI site) |
| Bahn33 | CGCGCGGCCGCGCCTTCATCGTCGTTAGAC | HRK1 in H99-right flanking primer (NotI site) |
| Bahn14 | CGAGAAGAATACAACGCAGTAG | HRK1 sequencing primer 1 |
| Bahn15 | CCGTTCTCAACAATCTCGTC | HRK1 sequencing primer 2 |
| Bahn16 | GCTGTATGTTTTGGTGAACC | HRK1 sequencing primer 3 |
| Bahn17 | CGGTAACAAGCATCTGAACG | HRK1 sequencing primer 4 |
| Bahn18 | TCACCTACTTTTCTGAGGCTC | HRK1 sequencing primer 5 |
| Bahn19 | AGAGTGCGAAGATTGACATC | HRK1 sequencing primer 6 |
| Bahn20 | CGTCAAATCCAAGAACATCGTG | HRK1 sequencing primer 7 |
| Bahn1086 | CTGCAGTTATTGGCCGGCTTGCACCTGCTG | HRK1 probe primer 1 for northern blot |
| Bahn1087 | AACAACATCGCCGCAATG | HRK1 probe primer 2 for northern blot |
| Bahn678 | TTCAGGGAACTTGGGAACAGC | ERG11 probe primer 1for northern blot |
| Bahn1598 | CAGGAGCAGAAACAAAAGC | ERG11 probe primer 2 for northern blot |
| Bahn2876 | CTCCTCTCCTTGCTTCTATTC | HRK1 qRT-PCR primer 1 |
| Bahn2877 | CGATGAACAGCGTAAGTAACG | HRK1 qRT-PCR primer |
|  |  |  |
